# Transvaginal Isthmocele Repair With Temporary Occlusion of Uterine Vessels in Caesarean Scar Pregnancy

**DOI:** 10.7759/cureus.54899

**Published:** 2024-02-25

**Authors:** Shubhada Jajoo, Suyash Naval, Rucha Naval

**Affiliations:** 1 Obstetrics and Gynaecology, Jawaharlal Nehru Medical College, Datta Meghe Institute of Higher Education and Research, Wardha, IND; 2 Laparoscopic Surgery, Naval Multispeciality Hospital, Jalgaon, IND

**Keywords:** p foley catheter, vaginal route, uterine vessels occlusion, isthmocele, caesarean

## Abstract

A uterine scar defect, or isthmocele, is one of the known complications of cesarean delivery. It can cause obstetric as well as gynecological problems. Diagnosis can be suspected based on complaints such as abnormal uterine bleeding, pelvic pain, dysmenorrhea, and subfertility. It can be investigated by transvaginal ultrasound and MRI hysteroscopy. A hysteroscopy gives a confirmatory diagnosis. Isthmoplasty may be offered to avoid future obstetric complications and treat symptoms. In the present case report, a patient with prolonged postmenstrual dark-colored spotting underwent isthmocele repair by a procedure that could be unique, which is transvaginal isthmocele repair with temporary occlusion of uterine vessels. This procedure offers efficacy, safety, good outcomes, and prospects. Cesarean scar pregnancy (CSP) is a rare but potentially serious complication of cesarean section deliveries. We describe the efficacy, safety, outcomes, and prospects of transvaginal Isthamocele repair with temporary occlusion of uterine vessels to manage CSP.

## Introduction

Cesarean section (CS), a prevalent mode of delivery, has witnessed a global increase in incidence in recent years [1]. Complications may arise, with Isthamocele, also known as cesarean scar defect, being one such concern [2]. Its prevalence on transvaginal sonography (TVS) examinations is 36.2% [3]. While often asymptomatic, esthamocele manifests as a pouch or defect in the anterior uterine wall at the cesarean scar site, potentially leading to significant obstetric and gynecologic morbidity [4].

Cesarean scar pregnancy (CSP) presents a distinctive challenge for both patients and healthcare providers. Detection methods include ultrasound, sonohysterography, and MRI [5]. Hysteroscopy, offering direct visualization, is a valuable tool for confirming Isthamocele [6]. The gold standard for Isthamocele management is surgical excision, which can be achieved through hysteroscopic resection, laparoscopic resection, or repair via a vaginal approach. The primary objective is to attain the full thickness of the myometrium.

In this context, we present a novel approach to transvaginal Isthamocele repair, incorporating temporary occlusion of uterine vessels as an intervention for women with cesarean scar pregnancies. The escalating incidence of CSP and its associated risks, such as uterine rupture and abnormal placentation, have compelled clinicians to explore innovative techniques. These techniques not only address immediate concerns related to pregnancy but also aim to mitigate the long-term sequelae associated with Isthamocele formation.

Our evaluation encompasses this proposed intervention's efficacy, safety, and outcomes, emphasizing its role in relieving symptoms of abnormal uterine bleeding, preserving fertility, and ensuring optimal maternal health. By amalgamating transvaginal repair techniques with the temporary occlusion of uterine vessels, our case report seeks to contribute valuable insights to the dynamic field of gynecological surgery.

## Case presentation

**Figure 1:**
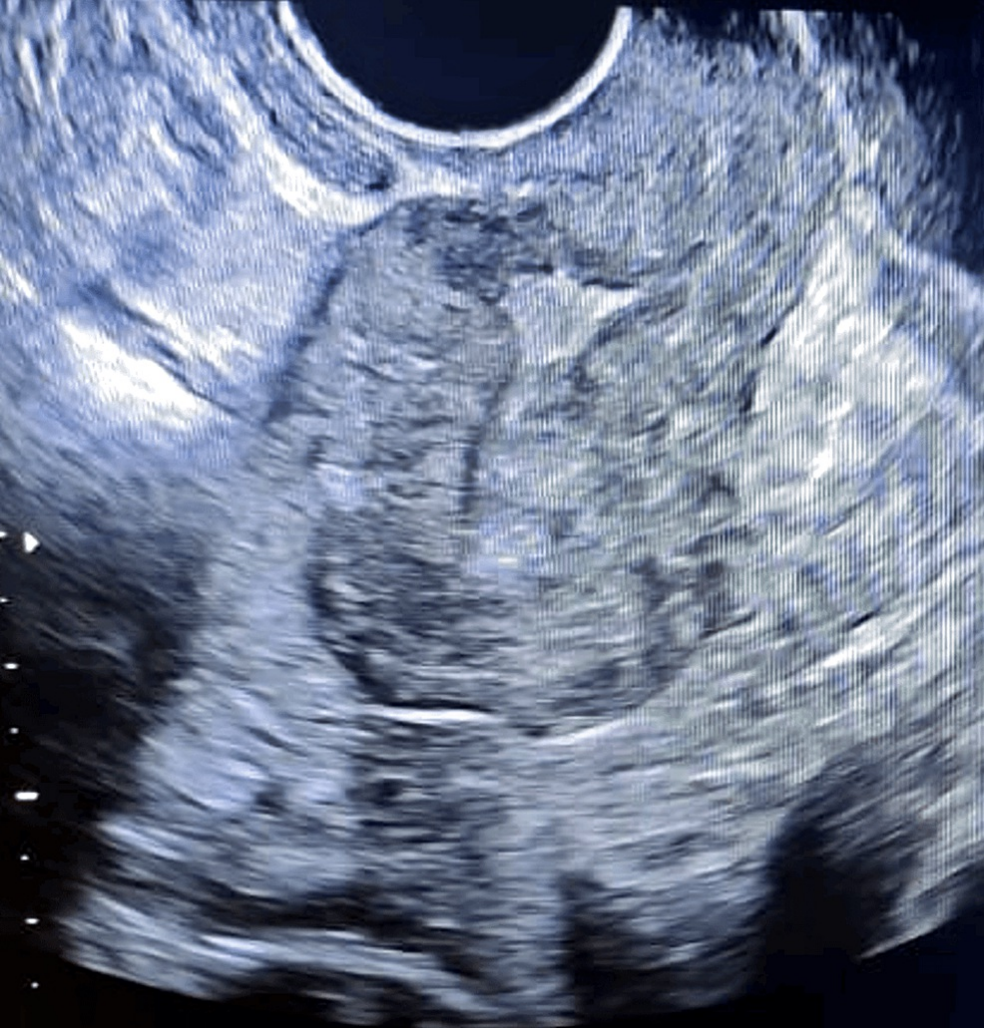
Showing ultrasonography preoperative Isthmocele

A 29-year-old woman with a history of previous cesarean section delivery presented with postmenstrual dark spotting and a desire for further fertility. Transvaginal ultrasonography revealed normal findings except for the presence of an Isthamocele, measuring 4.8 mm in breadth, 2.8 mm in length, and residual myometrial thickness of 2.9 mm and adjacent myometrial thickness of 4.4 mm at the previous cesarean scar site (Figure 1). The decision was made to perform transvaginal repair due to the ischiocele’s proximity to the anterior fornix.

**Figure 2:**
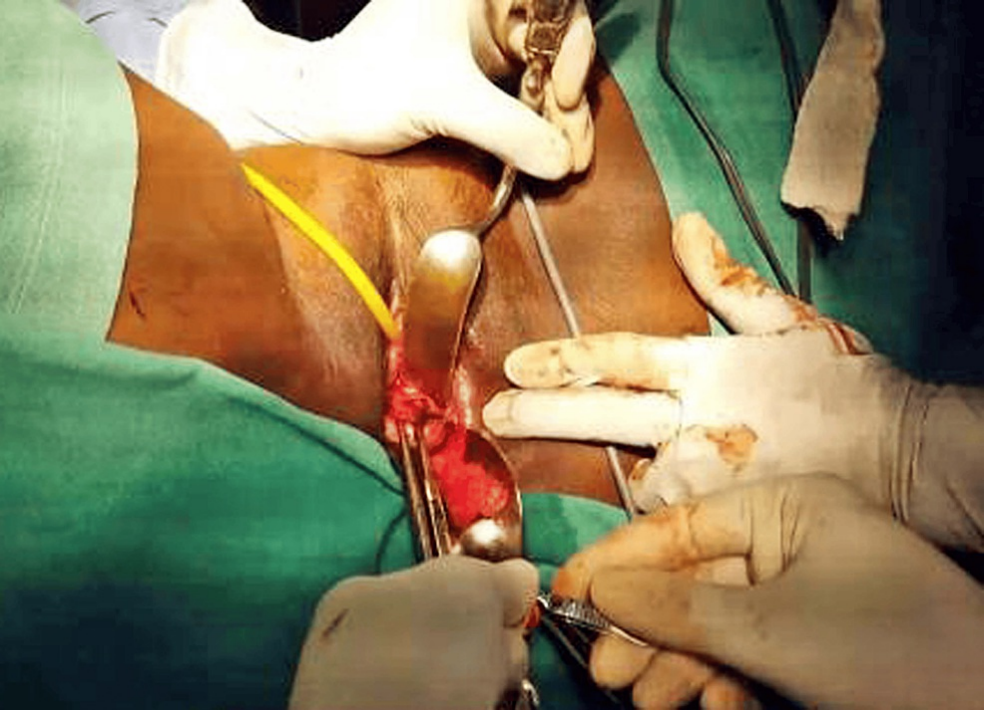
Shows temporary occlusion of uterine vessels with Buldogs vascular clamps to minimize blood loss

The surgical procedure began with the instillation of dilute adrenaline saline (1:200,000) in the cervicovaginal space, followed by an anterior colpotomy. Bladder dissection occurred in the lateral window in the Shirish Seth space, isolating the uterine arteries. Temporary occlusion of the uterine vessels was achieved using a small bulldog clamp after skeletonizing them (Figure 2).

**Figure 3:**
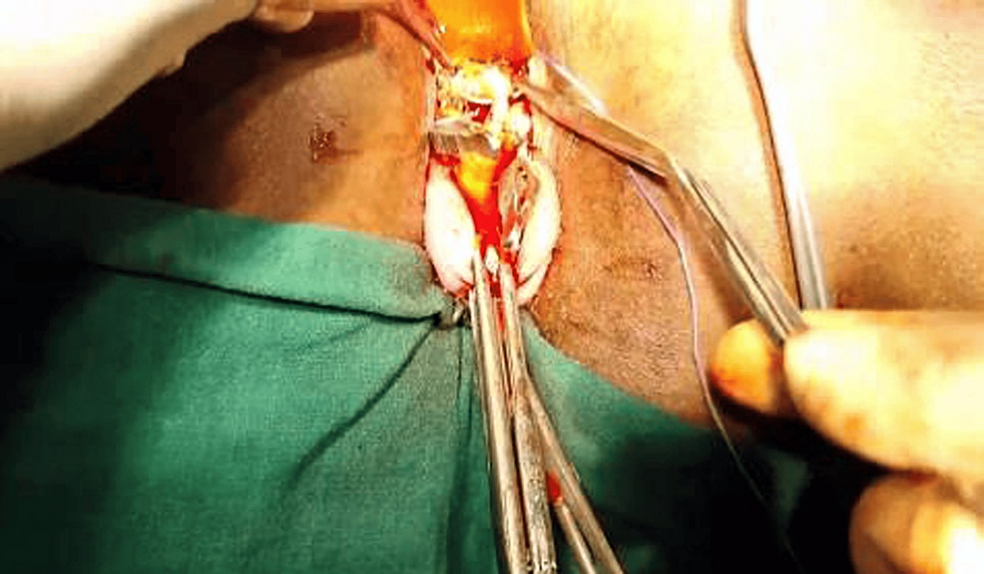
After incision, the Isthamocele defect was repaired in two layers to achieve good strength of the scar postoperatively

Subsequent hysteroscopic evaluation of the uterine cavity was conducted, creating transillumination at the Isthamocele level. This facilitated precise localization under hysteroscopic vision and transillumination. The Isthamocele was incised, revealing a gush of fluid through the hysteroscope. After widening the incision, the entire defect was palpated to understand its limits. Edges were refreshed, and the defect was repaired with polyglactin 1-0 in double-layer suturing, interrupted for the first layer and continuous for the second layer (Figure 3).

**Figure 4:**
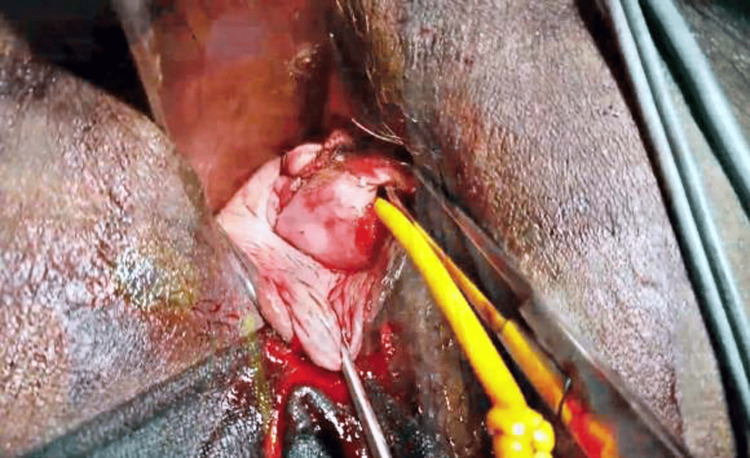
Shows pediatric Foley's catheter is inserted in the uterine cavity to prevent adhesions and stenosis

Bulldog clamps were subsequently removed from the uterine vessels. A pediatric Foley catheter was introduced and inflated with 2 ml of normal saline. It was kept for three days. Its placement at the isthmocele site was checked as a routine afterward with TVS. This procedure is done to prevent adhesions (Figure 4).

**Figure 5:**
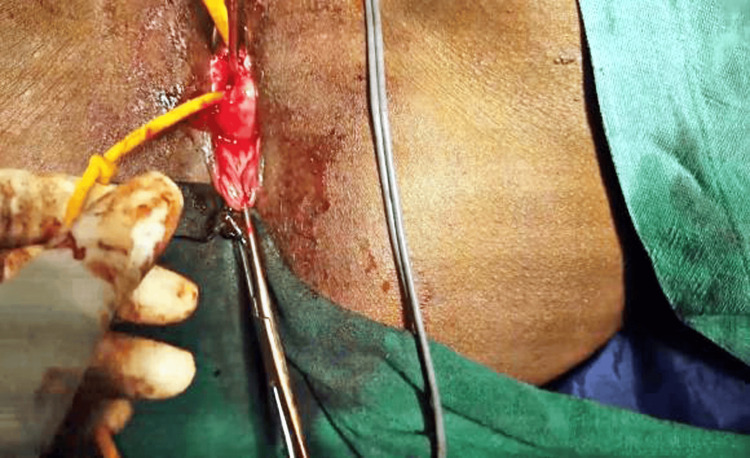
Shows complete repair of defect in two layers

Finally, the vaginal incision was closed with polyglactin 2-0, completing the repair of the defect, and the overall vaginal repair was done. The drain was not inserted as good hemostasis was achieved (Figure 5).

**Figure 6:**
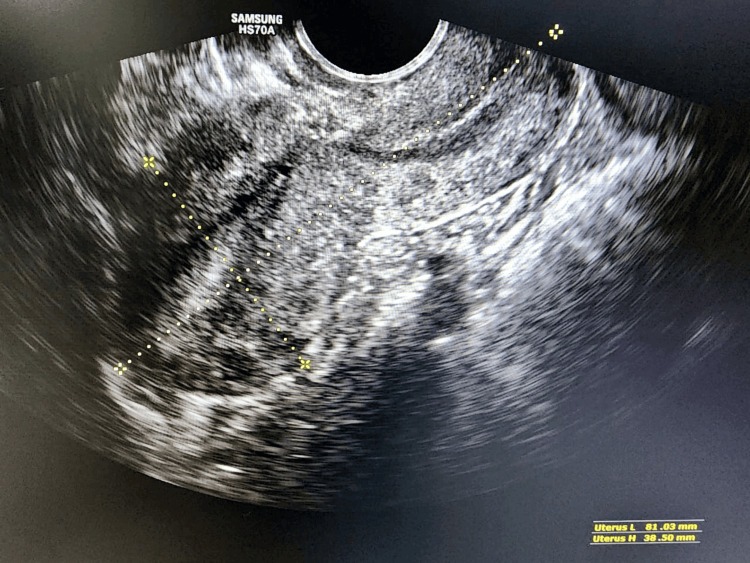
Showing ultrasonography post operative Isthmocele

After three months, the patient was reviewed with sonography, and the report showed complete healing. There was no residual Isthmocele; the post-USG showed healing of the scar (Figure 6). The preoperative complaints of the patient were resolved. The patient was advised to review after three months if there was no pregnancy or if she missed her menses at any time. 

## Discussion

**Figure 7:**
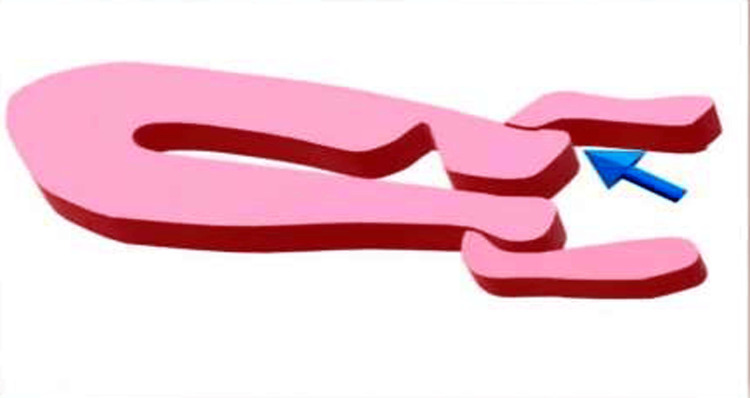
Shows a diagrammatic representation of a scar defect Figure credits: Dr. Shubhada Jajoo

Isthmocele is a manifestation of a pouch or defect that develops in the anterior uterine wall at the caesarean section scar site, potentially leading to significant obstetric and gynecological complications (Figure 7).

Isthmocele and associated gynecological and obstetric problems have increased globally, which is alarming. Pre-pregnancy surgical repair of caesarean scar defects is not required in all patients. Still, for symptomatic patients and those who want to conceive again, prior surgical repair may be a safe option [7]. Because of the two-child policy, if the patient has undergone a first caesarean section, caesarean scar defect (CSD) and its long-term complications have recently captured the attention of more gynecologists. Patients with Isthmocele may face problems with infertility because accumulated blood can degrade the quality of sperm and cervical mucous [8]. Operative methods in symptomatic Isthmocele advocated include hysteroscopy, laparoscopy, and vaginal repair [8,9]. Studies have indicated that amongst all surgical methods, vaginal repair for CSD is a minimally invasive procedure; it allows good exposure and accurate resection and repair of full-thickness myometrium [10].

In this case, where repair is done transvaginally aided with illumination by hysteroscopy and temporary occlusion of uterine vessels led to accurate visualization of scar defect, its optimum resection and good repair with minimal blood loss [11]. This seems to be a novel approach to the repair of CSD with promising results. The main advantages of this method are the least invasive, short operating time, easy to learn, similar efficacy and recovery compared to laparoscopy, and low cost. After surgery, the six-month interval is advocated before attempting the next pregnancy [12]. This approach represents a suitable choice for the surgical management of CSD. The treatment of isthmocele ranges from clinical management with oral contraceptives and surgical treatment as hysterectomy to sparing techniques, including hysteroscopic, laparoscopic, laparotomic or transvaginal procedures limited to the defect side [13]. The decision of treatment should take into consideration the size of the defect, the presence of symptoms, secondary infertility, and plan of pregnancy [14].

## Conclusions

In conclusion, among all the techniques advocating transvaginal Isthamocele repair with temporary occlusion of uterine vessels, the surgical management of caesarean scar pregnancies is justified. This approach is minimally invasive, ensures swift procedure, is user-friendly, entails fewer adverse events, is cost-effective, and demonstrates efficacy comparable to laparoscopy. Consequently, it can be deemed a preferred choice in clinical practice. Our case report offers valuable insights into the dynamic field of gynecological surgery, emphasizing the potential of this method to enhance patient outcomes and streamline healthcare resource utilization.
